# Dry needling in a manual physiotherapy and therapeutic exercise protocol for patients with chronic mechanical shoulder pain of unspecific origin: a protocol for a randomized control trial

**DOI:** 10.1186/s12891-017-1746-3

**Published:** 2017-09-18

**Authors:** Emma Tejera-Falcón, Nuria del Carmen Toledo-Martel, Francisco Manuel Sosa-Medina, Fátima Santana-González, Miriam del Pino Quintana-de la Fe, Tomás Gallego-Izquierdo, Daniel Pecos-Martín

**Affiliations:** 1 Private practice, Gran Canaria, Spain; 2 Department of Physical Therapy and Pain Group, Universidad de Alcalá, Alcalá de Henares, Spain; 3 Facultad de Enfermería y Fisioterapia, Universidad de Alcalá (Spain), Campus Científico-Tecnológico: CRTA. Madrid – Barcelona, km.33,600, Alcalá de Henares, Spain

**Keywords:** Dry needling, Shoulder pain, Physical therapy, Therapeutic exercise, Myofascial trigger point

## Abstract

**Background:**

Shoulder pain of musculoskeletal origin is the main cause of upper limb pain of non-traumatic origin. Despite being one of the most common reasons for consultation, there is no established protocol for treatment due to the complexity of its etiology. However, it has been shown that the presence of myofascial trigger points on the shoulder muscles is a common condition associated with patients suffering from shoulder pain. This protocol has been created which describes the design of a randomized controlled trial to evaluate the effectiveness of the inclusion of dry needling (DN) within a protocol of manual physiotherapy and therapeutic exercise in the treatment of chronic shoulder pain of unspecific origin.

**Methods:**

Thirty-six participants aged 18–65 years will be recruited having mechanical chronic shoulder pain on unspecific origin and meeting the inclusion criteria. These will be randomized to one of two interventions, (i) DN, manual physiotherapy and therapeutic exercise or (ii) sham DN, manual physiotherapy and therapeutic exercise. The protocol will cover 6 weeks of treatment, with a 6-month follow-up. Our main outcome measure will be the Visual Analogue Scale for pain.

**Discussion:**

This is the first study to combine the use of DN, manual physiotherapy and an exercise program with a 6-month follow-up, thus becoming a new contribution to the treatment of chronic shoulder pain, while new lines of research may be established to help determine the effects of DN on chronic shoulder pain and the frequency and proper dosage.

**Trial registrations:**

International Standard Randomized Controlled Trial Number Register: ISRCTN30604244 (http://www.controlled-trials.com) 29 June 2016.

## Background

Shoulder pain is a common musculoskeletal problem, with an annual prevalence of 20 to 50%, being the main cause of non-traumatic upper limb pain. It presents a high chronicity and recurrence [1] and the symptoms persist for 6 to 12 months in 40 to 50% of patients [2]. However, there is no standard for the clinical definition of shoulder pain [3].

Clinical trials tend to use the term nonspecific shoulder pain due to the lack of agreement on diagnostic criteria, lack of specificity of clinical evidence, coexistence of multiple shoulder pathologies and lack of any diagnostic test that is considered a “gold standard” [4]. The most common signs and symptoms are localized in the deltoid, forearm and shoulder region, presenting shoulder stiffness and limited range of motion [5] which restrict daily living activities [6].

Shoulder Impingement Syndrome is the most common diagnosis in primary care [7–9] and although it is believed to be the cause of shoulder pain, there is no solid evidence to support this. Furthermore, the presence of calcifications, acromial bone spurs, subacromial swelling, degenerative rupture of the rotator cuff, tendon inflammation and signs of degeneration are prevalent in both healthy subjects and in subjects with shoulder pain [10–12] so its diagnosis alone would not justify the presence of symptoms.

Moreover, it has been shown that the presence of myofascial trigger points (MTrPs) in the shoulder muscles is a common condition in patients with shoulder pain [3], and may cause pain during muscle stretching, contraction or compression. These MTrPs are hyperirritable points in taut bands of skeletal muscle and are painful to compression, producing motor dysfunction and referred pain [13].

As many as 17 muscles are known to reproduce similar symptoms to those of other painful shoulder syndromes, including pain at rest, upon movement and sleep disorders [1, 13, 14]. Therefore, the presence of MTrPs has been suggested as an alternative explanation for shoulder pain, regardless of the presence of subacromial disorders [1, 14].

Treatment of shoulder pain usually begins with conservative therapies such as rest, physical therapy, anti-inflammatory drugs (NSAIDs) and corticosteroid injections [15, 16]. However, current studies have shown the benefits of a multimodal treatment for shoulder pain including techniques such as dry needling, stretching, manual therapy, mobilization techniques, applying cold, home exercise, ischemic compression of MTrPs and ergonomic recommendations [1, 17].

The choice of treatment is often subjective and depends on the therapist’s skill and training, while therapeutic exercise programmes in combination with manual therapy techniques often show good results [5, 18]. A recent meta-analysis by Kietrys et al. [5] recommends using dry needling, compared against placebo, to reduce pain, following treatment, and at 4 weeks follow-up in patients with Myofascial Pain Syndrome of the Upper Quadrant. However, new studies to support this recommendation are required.

It has been suggested that the inclusion of dry needling, in a single session, within a multimodal physiotherapy programme for patients with post-surgery shoulder pain, produces improvements in on and range of motion [19]. However, we have not found similar studies in patients with chronic shoulder pain of unspecific origin.

Therefore, the main aim of this study is to determine the effectiveness of including dry needling in a manual physiotherapy and therapeutic exercise programme, for the treatment of chronic shoulder pain of unspecific origin. Our hypothesis is that by including a single session of dry needling in a manual physiotherapy and therapeutic exercise programme, treatment outcomes improve, reducing symptoms and improving function in patients with shoulder pain.

## Methods

### Design

Study protocol for a randomized controlled parallel group single-blind trial. This study was registered with the International Standard Randomized Controlled Trial Number (ISRCTN30604244) and complies with the recommendations of The SPIRIT 2013 Statement.

Participants will be randomized to receive dry needling, either real or sham. Allocation to either group, namely real or sham dry needling, will be achieved through a computer-generated sequence of random numbers. The allocation sequence is created and carried out by a non-interventionist physiotherapist, in charge of telephone screening and of handling the data obtained in the various assessment sessions.

**Fig. 1:**
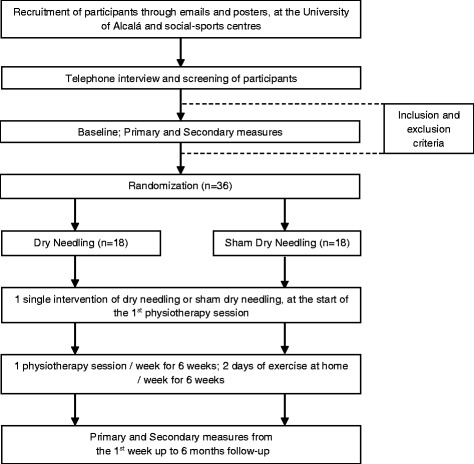
Study Flowchart. Stages of the intervention protocol

Figure 1 shows a flowchart of the progress of the various stages of this test. This study was approved by the Committee on Human Research, Universidad de Alcalá (Reference: CISM/HU/2015/19).

### Participants

From the town of Alcalá de Henares (Madrid), aged between 18 and 65 years with chronic mechanical shoulder pain of unspecific origin, lasting at least 3 months and provided they give their consent after being informed about participation in the study. The recruitment of participants will be conducted via email for University of Alcalá workers and students and by placing posters in a number of social and sports centres around the city of Alcala de Henares, where information about the study and contact information will be provided. Subsequently, a first telephone interview will be conducted to clarify any doubts of the participants, and the first screening for inclusion in the study will be carried out.

Participants’ personal data will be numerically coded and stored in a computer database, which may only be accessed to by the physiotherapist in charge of participant randomization and blinding.

Informed consent, as well as all study information will be emailed to the participants. Subsequently, prior to data collection and baseline measurement, participants should sign the informed consent, which could be revoked at any time during the intervention.

Inclusion criteria:

- Chronic mechanical shoulder pain of unspecific origin lasting at least 3 months.
- Aged between 18 and 65 years.
- Presence of active trigger points or areas of mechanical hypersensitivity in the muscles to be treated (upper trapezius, infraspinatus, subscapularis and middle deltoids) with pain reproduced in one or more muscles.

Participants will be examined for the presence of active trigger points in the muscles selected by a clinician with more than 10 years of experience in the treatment of MTrPs. Diagnosis of MTrPs will be determined by the presence of the following criteria: [1] hypersensitive point in a palpable taut band, [2] visible or palpable local spasm in response to MTrP palpation, and [3] reproduction of referred pain by palpation of the sensitive spot. These criteria have shown good interexaminer reliability (κ, kappa between 0.84 to 0.88) when applied by an experienced evaluator [20].

Exclusion criteria:

- Previous surgery on the shoulder.
- Previous history of shoulder dislocations.
- Whiplash.
- Cervical radiculopathy.
- Diagnosis of fibromyalgia.
- Diabetes.
- Needle phobia or any contraindication to dry needling (e.g. anticoagulants or psychiatric disorders).
- Bilateral shoulder pain.
- Pregnancy.
- Having received dry needling in the last 6 months.
- Currently receiving other physiotherapy treatment for shoulder pain.

### Research team

This study will involve 7 physiotherapists; 1 Clinical physiotherapist with more than 10 years of experience in the use of dry needling for MTrP treatment, 2 evaluator physiotherapists and 2 physiotherapists responsible for carrying out the manual physiotherapy and therapeutic exercise treatment of the participants, one physiotherapist responsible for the interview, initial screening and randomization of participants, and one physiotherapist who will perform the statistical analysis.

### Sample size

The pain variable is chosen as the primary measurement of the study results. An effect size TE = 0.25 will be considered [21]. A correlation between repeated measures of 0.5 shall be assumed. Assuming the performance of 6 measurements -basal and a week after the dry needling, and post-treatment: at one week, at one month, at 3 months and at 6 months- in two treatment groups, sphericity correction will be determined at 0.5. With a statistical power of 0.95, with an alpha level of 0.05, a total sample size of 28 patients is estimated, and taking into account 25% for losses, a total of 36 patients needs to be reached, being 18 in both groups, using the Software Gpower 3.0.18 [22].

### Randomization and blinding

At baseline, the non-interventionist physiotherapist, through the Random Allocation Software, will randomize by permuted blocks [4, 8], the participation of the 5 intervening physiotherapists with respect to a sample of 36 participants. The physiotherapist in charge of performing dry needling –which may be A (experimental) or B (control)- and 2 groups of 2 physiotherapists, G1 and G2, with an evaluator and a therapist in each group. 4 possible treatment groups (AG1, AG2, BG1, BG2) will be obtained evenly in each randomization block.

Participants who successfully pass the phone screening will be assigned a number on the randomization table in order of inclusion in the study; the treatment group (A or B) and physiotherapist group (G1 or G2) in which they will be included are specified. The physiotherapist responsible for carrying out the dry needling, will provide a list with the names of the participants, showing the type of intervention that each will receive (A or B).

Each participant will receive a single dry needling or sham dry needling intervention at the start of the first treatment session, conducted by a physiotherapist with more than 10 years of experience in MTrP treatment with dry needling. Said physiotherapist will be blinded to the baseline data of participants. On the other hand, both the physiotherapists who evaluate the participants and those who perform manual physiotherapy treatment and therapeutic exercise, will be unaware of the group of each participant.

In addition, data collected during the assessment of participants will not be revealed to the physiotherapists who perform the treatment, and participants will be instructed not to disclose their experience and information related to the first treatment session of dry needling.

Lastly, the physiotherapist responsible for the statistical analysis will be blinded. Once the intervention is finished he will receive a data table in excel with all the necessary data in binary code.

### Interventions

Participants included in this study will receive 6 treatment sessions (one per week), with a corresponding evaluation before the start of each treatment session. In addition, the participants after completing the last treatment session, will receive post-treatment assessments; at one week, at one month, at 3 months and at 6 months.

### Dry needling of myofascial trigger points

To perform the dry needling procedure, patients should be placed on: their healthy or good side for infraspinatus and deltoids muscle; supine decubitus for subscapularis muscle; and prone decubitus for upper trapezius muscle. The physiotherapist will treat each MTrP that have previously been located in the muscles: upper trapezius, infraspinatus, subscapularis and middle deltoid.

After this, a 0.30x40mm monofilament needle (AGUPUNT, APS®) will be inserted towards the muscle mass. Said needle should be inserted into and extracted from the muscle using the “fast in and fast out” technique [23]. Needle insertion will be repeated 12 times in each muscle, the dry needling procedure being similar to that used by Hong [23, 24]. At most one MTrP (the one producing most pain) in each of the muscles will be needled. If no MTrP is found during muscle palpation, the needling procedure will not be performed on that muscle.

Meanwhile, the control group will receive the same treatment but using 0.30x30mm sham needles (Streitberger Placebo - needle®, Asiamed), which have been used before in another study [25].

Participants in both groups, will receive the same information about the sensations of dry needling procedure. They will be informed, at the beginning of the technique, that they may or may not feel the introduction of a needle through the skin. This will be related to the area of the body where the puncture is performed and according to the sensitivity of the subject. Afterwards, they will feel as the physiotherapist manipulates the needle with his hand repeatedly. In addition, participants should communicate to the physiotherapist the sensations they notice during the performance of the technique.

### Manual physiotherapy and therapeutic exercise programme

Previous studies have shown that the inclusion of manual physical therapy in an exercise programme is more effective than exercise alone in reducing pain, increasing range of motion, strength and functionality [26, 27]. However, evidence has failed to show if an exercise mode is better than another, or which are the optimal frequencies or intensities thereof [27].

Treatment sessions will be implemented by two physiotherapists and divided into two parts; the first part will be based on manually treating the affected shoulder and the second part will be based on supervised therapeutic exercise. In addition, participants will be prescribed therapeutic home exercises, twice weekly (alternate days).

Manual physiotherapy treatment will last approximately 45 min. Techniques used in previous studies have been chosen for the treatment protocol, such as trigger point pressure release technique [28], longitudinal massage [28], scapulohumeral mobilization [19, 29], glenohumeral mobilization [19, 30], glenohumeral gliding and gapping [31–33] and mobilizations with active movement [34, 35]. The description and dosage of the techniques are contained in Table 1.

**Table 1:** Physiotherapy intervention components

Treatment section	Dose
MTrP pressure realease technique On the most hyperalgesic point of the infraspinatus, upper trapezius, middle deltoids and subscapularis muscles. If there is no MTrP, the technique will be not applied to that muscle.	Twice on each point to be treated during 60 s and a 15 s rest.
Longitudinal massage Slow and deep massage along the muscle band of the hyperalgesic point treated above, with a tolerable pressure for the participant. If there is no hyperalgesic point, it will be applied to the entire muscle.	3 longitudinal sweeps across each taut band found, with an approximate duration of 20 s per sweep.
Scapular-humeral mobilizations Passive movements of ascent and descent, abduction and adduction, internal and external scapular rotation, scapular distraction movements and scapular circumduction of the affected shoulder.	10 repetitions of each movement, in the absence of pain or only slight discomfort tolerated by the participant. Participant in lateral decubitus of the healthy side.
Glenohumeral joint mobilizations Anterior and posterior passive movements of the glenohumeral joint. For the treatment position, the shoulder will be previously placed in passive abduction to end of ROM without pain.	2 sets of 20 anterior and posterior mobilizations. Participant supine.
Glenohumeral gapping and gliding Passive shoulder abduction to end range without pain. Subsequently, a technique of caudal gapping and gliding of the glenohumeral joint will be performed.	3 sets of 15 repetitions. Participant supine.
Mobilizations with active movement Slow and controlled active shoulder flexion movement to end range without pain. During the active movement, the physiotherapist will secure the scapula and will perform a posterolateral thrust on the humeral head.	3 sets of 5 repetitions in a sitting position. If there is pain, will find a plane of motion without pain or will change the position of the supporting hands.
Therapeutic exercise Performed during the treatment sessions and at home in 3 progressive stages, each lasting 2 weeks.	Sessions; once/week, 25 min with the physiotherapist’s supervision. At home; twice/week, 20 min.

The supervised therapeutic exercise will last approximately 25 min, which will be conducted in 3 progressive stages, 2 weeks each stage. Exercises used in previous studies have been selected such as scapulohumeral stabilization exercises [26, 29, 36], anterior [26] and posterior [37] flexibilization of the joint capsule, Codman exercises [26], proprioception, active and self-paced shoulder exercises [26], full-can exercise [26, 38], strengthening of the rotator cuff and scapular muscles [26, 39, 40]. The description and dosage of the exercises are set out in Table 2.

**Table 2:** Description of the therapeutic exercises

Name and description	Dose	Weeks performed
Non-weight-bearing scapulohumeral stabilization exercise (CKC) Standing; 90° shoulder flexion and hands resting on the wall. Slow scapular movements down towards the midline of the back. The physiotherapist will give tactile stimuli for activation of the lower trapezius and serratus anterior. In the 3rd and 4th weeks, more weight will be placed on the arms by tilting the trunk.	3 sets × 8 reps 4 sets × 5 reps	1st & 2nd 3rd & 4th
Scapulohumeral stabilization (OKC) Standing; arms relaxed. Combined movements of lifting, retropulsion and lowering of both shoulders. During retropulsion and lowering should, shoulder blades should be stabilized in the dorsal midline.	3 sets × 10 reps	1st & 2nd
Anterior and posterior flexibilization of the joint capsule Anterior flexibilization; standing with hand and forearm leaning on a doorframe, the ipsilateral leg forward and the trunk tilted forward until a feeling an anterior stretching sensation, without pain. Posterior flexibilization, lateral decubitus of the affected side, shoulder and elbow at 90 degrees of flexion. From this position, the arm is brought towards the stretcher with the help of the other hand, until feeling posterolateral stretching without pain.	30 s × 3 times (each stretch)	1st & 2nd
Codman’s exercise Standing; trunk tilted forward with the unaffected arm supported on a high surface. Affected arm should be relaxed and fall freely. With the help of the unaffected hand, perform passive swing movements, avoiding muscle activation of the affected arm.	3 sets × 25 reps	1st & 2nd
Proprioception with a fitball Sitting, with the affected arm stretched and continuously supported on the fitball, perform side, anterior and diagonal movements, helping by tilting the trunk. 2nd set with closed eyes. 3rd set with destabilization thrusts on the fitball.	2 reps each movement. Until completion of 1 set.	1st & 2nd
Self-paced shoulder flexion and abduction Standing; hands apart at shoulder level, holding a wooden stick. Flexion; arms extended to AROM without pain, in a controlled manner. Abduction (affected side); arms extended to end range without pain arms, in a controlled manner. If there is discomfort or lack of strength during the exercises, complete the ROM with the help of the other arm. On the descent of both movements, scapular control will be required.	3 sets × 8 reps (each movement)	3rd & 4th
Active shoulder flexion using a fitball Standing; hands resting on a fitball against the wall and leaning forward. Active flexion movements alternating both arms, rolling the fitball along the wall to reach the maximum flexion of the affected shoulder without pain.	3 sets × 6 reps	3rd & 4th
Full-can Standing; arms stretched at 90° abduction and 30° horizontal flexion. External rotation movements of the shoulder and lowering of the scapula.	4 sets × 10 reps	3rd & 4th
Scapulohumeral stabilization using weights (OKC) Standing; leaning against the wall. Horizontal bending movements (from 90° to 0°) holding a dumbbell of 0.5 or 1 kg according tolerance in each hand. During movement take both scapulae to the midline of the back in a controlled manner.	3 sets × 8 reps	5th & 6th
Strengthening of the rotator cuff and scapular muscles (Theraband) Flexion, extension and abduction; in the standing position, with the arm extended and holding the band with the hand of the affected arm and holding the other end with the ipsilateral foot, will perform the exercises: [1] flexion, [2] extension and [3] abduction to end AROM without pain. During the abduction return movement, the patient will be instructed to control the internal rotation and scapular adduction movement. External and Internal rotation; in the standing position, with a towel between the body and the affected arm, shoulder at 0° flexion and elbow at 90° flexion, gripping the band with the hand of the affected arm and securing the other end to the doorknob, will perform the exercises: [4] external rotation and [5] internal rotation to AROM without pain.	Flex. & Abd.; 3 sets × 8 reps // Ext., Rot. Ext. & Int.; 3 sets × 10 reps	5th & 6th

Therapeutic exercises at home will last 15 to 20 min or so. Exercises will perform twice per week (alternate days) and for which the physiotherapist will provide the guidelines and training in the treatment sessions.

The exercises will comprise 3 progressive stages as mentioned above, following an information sheet with the relevant instructions for each exercise. In each treatment session participants will be asked about the exercises performed at home, to learn about any difficulties encountered and make the necessary corrections. Participants will be instructed to continue performing therapeutic exercises at home once the intervention is completed and until the last evaluation (at 6 months post-treatment).

Participants will not receive any physiotherapy or medical treatment for shoulder pain during the study intervention. Should any adverse event occur during the execution of the dry needling, or at any time during the intervention in the study, participants will discontinue participation.

### Evaluations

During the baseline measurement, the following descriptive characteristics will be collected: (i) gender, (II) age, (III) weight, (IV) height (V) dominant hand, (VI) current occupation, (VII) sport. In addition, the following information concerning the affected shoulder will be collected: (I) painful side, (II) duration of pain, (III) prior illness, (IV) taking medication. The chronology of the evaluation of the primary and secondary outcome measures is shown in Table 3.

**Table 3:** Chronology of primary and secondary outcome measures

Results										
	Baseline Week 1	Week 2	Week 3	Week 4	Week 5	Week 6	1 week post	1 month post	3 months post	6 months post
Primary										
VAS	✔	✔	✔	✔	✔	✔	✔	✔	✔	✔
DASH	✔						✔	✔	✔	✔
Secondary										
Dynamometry	✔	✔					✔	✔	✔	✔
Goniometry	✔	✔					✔	✔	✔	✔
Algometry	✔	✔	✔				✔	✔	✔	✔

### Primary outcome measures

The main result of this study will be the intensity of shoulder pain measured by ***Visual Analogue Scale (VAS) for pain***. Participants will mark the intensity of their pain on the VAS, consisting of a 100-mm long horizontal line, which is anchored by the classifications of “no pain” at the left end (score 0) and “worst pain imaginable” rightmost (score 10), asking to the participant for the most intense pain episode perceived meanwhile doing daily activity. The VAS has demonstrated the ability to detect changes in pain, establishing a minimal clinically significant difference at 13 mm [41, 42]. And in patients treated for rotator cuff disease a difference at 14 mm [43].

### Secondary outcome measures

1. ***Disabilities of Arm, Shoulder and Hand (DASHe), Spanish version.*** It will be used to determine the disability rate related to shoulder pain. The DASHe is a self-administered questionnaire consisting of a central body of 30 items and 2 optional modules, each with 4 items, intended to measure the impact of injury of the upper limb by playing musical instruments and performing sport or work. Each item is scored 1 to 5, with increasing values depending on the severity of symptoms. The score of the items are summed to obtain a total score, which can range from 30 to 150 points, which is converted to a scale from 0 (best possible score) to 100 (worst possible score).
  Optional modules, if any, are scored separately by the same method. The limit to invalidate the DASHe questionnaire is 4 or more unanswered questions. DASHe questionnaire allows assessing the perceived disability for the patient to perform various activities, including activities of daily life and symptoms such as pain, stiffness and loss of strength [44]. The minimally significant clinical difference in the DASHe for musculoskeletal upper limb problems in adults is 10.2 [45].
2. ***Pressure Pain Threshold (PPT).*** Performed with a pressure algometer (Baseline® 30 kg) on the point of greatest mechanical hyperalgesia of the patient’s shoulder, on the spinous process of C7 and on the area of greatest mechanical hyperalgesia of the tibialis anterior. PPT is defined as the amount of pressure applied on the point to be assessed until a painful sensation appears. Three measurements will be carried out and the average will be calculated, to be used for data analysis. 30 s rest will be left between measurements. C7 and tibialis anterior measurements will be used to determine the effect of the central modulation of shoulder pain [28, 46].
  It has been established that algometry is a highly reliable technique for PPT measurements when the examiners are well trained. Chesterton et al. [47] showed that changes of more than 17.39 N/cm2 (1.77 kg/cm2) can be considered with certainty to represent real change. Algometry has been used to measure the impact of manual therapy on the treatment of trigger points in cases of shoulder pain with a one month follow-up [28].
3. ***Shoulder range of motion***. It will be measured with a standard 18 cm plastic goniometer (Sammons Preston-Rolyan®). The following shoulder movements will be measured: flexion, extension, internal rotation, external rotation and abduction to end range without pain. Each movement will be measured three times, of which the average will be calculated, to be used in the analysis.
  The universal goniometer has shown good intraobserver reliability (intraclass correlation coefficient from 0.91 to 0.99), if consistent benchmarks are used [34], for flexion, extension, abduction and rotation [48]. A change of 6° to 11° is needed to be certain that there has been a real change in the goniometric measurements of the shoulder [34].
  Flexion will be measured with the participant in a sitting position with a strap around the abdomen and the back of the chair to limit trunk compensation. The goniometer axis will be aligned with the centre of the joint axis (inferior and lateral to the acromion). The fixed arm of the goniometer will follow the line of the trunk, and the mobile arm, parallel to the longitudinal axis of the humerus and it proceeds to actively raise the arm in the sagittal plane; with the thumb pointing upwards [34].
  For the extension movement, the participant will be placed prone; with the shoulder in the neutral position, elbow flexed 90° and the forearm in the neutral position. The goniometer axis will be placed at the midpoint of the lateral aspect of the glenohumeral joint. The fixed arm will be placed parallel to the patient’s trunk, and the mobile arm, parallel to the longitudinal axis of the humerus. And finally the arm will be actively extended in the sagittal plane [34].
  For the abduction movement, the participant will be placed in a sitting position with a strap around the abdomen and the back of the chair to limit trunk compensation. The goniometer axis will be located at the midpoint of the posterior side of the glenohumeral joint. The fixed arm will be placed parallel to the trunk, and the mobile arm, parallel to the longitudinal axis of the humerus. And finally the arm will be actively abducted in the frontal plane, with the thumb pointing upwards to allow the necessary external rotation [34].
  As for the internal rotation movement, it will be performed with the participant prone; 90° shoulder abduction, 90° elbow flexion and forearm in the neutral position. To avoid compensation, the thumb will be placed on the coracoid exerting pressure and the other fingers on the spine of the scapula, to control scapular rise. The goniometer axis will be aligned with the elbow olecranon. The fixed arm will stand upright, vertical to the floor and the mobile arm of the goniometer will be aligned along the midline of the forearm. Finally performing internal rotation [34].
  External rotation will be performed with the participant supine; hips and knees bent at 45°, 90° shoulder abduction, 90° elbow flexion and forearm in neutral. The goniometer axis will be aligned with the elbow olecranon. The fixed arm vertical to the floor and the mobile arm of the goniometer aligned along the midline of the forearm, finally performing external rotation [34].
4. ***Strength in shoulder movements***. It will be measured with a hand dynamometer (microFET2® Hoggan Scientific LLC). The measurements will be taken in pounds and converted to kilograms-force. Strength in the following movements will be measured: flexion, abduction, external rotation and internal rotation.
  For measurements of isometric force in ER (external rotation) and IR (internal rotation), it has been found that the intraclass correlation coefficient (ICC) is 0.93 to 0.99. The minimum detectable change varies from 7.87 N (External Rotation) to 22.11 N (Internal Rotation) [48].
  The physiotherapist should stabilize manually or with straps the upper arm, shoulder, scapula and trunk, while performing the tests.
  Measuring the strength in flexion will be conducted with the participant in a sitting position with the shoulder at 45° of flexion and the elbow extended; the Dynamometer will be placed above the lateral epicondyle. The trunk will be strapped to avoid compensation [49]. Abduction will be carried out with the participant in a sitting position with 90° shoulder abduction, 90° elbow flexion and forearm neutral, positioning the dynamometer just proximal to the lateral epicondyle [50].
  Measurement of the internal rotation will be carried out with the participant supine; shoulder abduction, 90° elbow flexion and forearm neutral. The dynamometer will rest on the ventral side of the forearm, 2 cm proximal to the styloid process [48].
  For the measurement of external rotation, the participant supine; 90° shoulder abduction, 90° elbow flexion and forearm neutral. The dynamometer will rest on the back of the forearm, 2 cm proximal to the styloid process [48].
  While performing the baseline measurement, data for secondary outcome measures of the participant’s healthy side will also be collected in order to establish whether there is a baseline difference between the two sides before starting the intervention.

### Physiotherapist training

For the assessment and treatment, a series of training stages prior to starting the study will be implemented, intended to protocolize the actions carried out in the study. In these training stages, treatment techniques and measuring will be practiced in order to reach a consensus among the physiotherapists involved. Moreover, an external observer will value similarities of interventions between physiotherapists.

### Statistical analysis

Data analysis will be carried out following the evaluation standards of the design of experimental studies with a control group. The experimental group (dry needling + manual physiotherapy and therapeutic exercise) will be compared with the control group (sham dry needling + manual physiotherapy and therapeutic exercise).

Data will be analysed using SPSS v.22 software for Windows. All statistical tests will be carried out considering a confidence interval of 95% (*p*-value <0.05) to determine the effectiveness of the 2 interventions by the method of intention to treat. Prior to statistical comparisons, all data will be analysed to determine the distribution of normality by the Shapiro-Wilk test. Subsequently, the homogeneity of the two intervention groups will be studied using Student’s t test for independent samples for data conforming to normal and the Mann-Whitney test for data that do not. The sex variable will be studied through Pearson χ2 or Fisher’s exact test, if the former cannot be used. Then a descriptive analysis of the data for the dependent variables will be performed. In these analyses, the mean and standard deviation (SD) for the dependent variables with normal distribution will be calculated. For variables that do not conform to normal, the data will be expressed with the median and first and third quartiles.

For the sex variable, frequencies are used. The existence of differences within each group will be determined, taking into account each group in isolation, between the different measurements (baseline and one week after dry needling; and post-treatment: at one week, at one month, at 3 months and at 6 months) in each of the study variables. In the VAS variable, measurements will also be taken in the treatment sessions; 2nd week, 3rd week, 4th week, 5th week, 6th week, using ANOVA for repeated measures, supplemented with simple and Helmert-type contrasts for variables that follow normal or Friedman’s ANOVA test, supplemented by Dunn’s multiple comparison test for those variables that will not conform to normal. ANCOVA will be performed to see the effect through different analyzes of the results and to intensify statistical power of the study. For this, the existence of a linear relationship and the homogeneity between the crude and basal scores of the dependent variables will be previously studied.

For comparison between groups, a variable that we define as “difference” will be found; for each dependent variable, by subtracting the baseline measurement - at one week following needling, baseline - post 1 week, baseline - post 1 month, baseline - post 3 months and baseline - post 6 months (for the DASHe variable, the measurement will not be collected at one week after needling).

For the VAS variable, the “difference” variables will be found between baseline - week 2, week 3, week 4, week 5, week 6. To determine differences in dependent variables between the two intervention groups, the Student t test will be applied for related samples in variables whose data follow normal, in this case the effect size will be calculated according to the formula d = 2 t/√g. In the case of variables whose data do not follow normal, an analysis by the Mann-Whitney test will be performed and the effect size will be estimated according to Grissom procedures according to the formula.

## Discussion

This protocol will be carried out for a randomized single-blind clinical trial, in order to investigate whether the inclusion of dry needling in a manual physiotherapy and therapeutic exercise programme has a greater effect in reducing pain and disability in subjects with chronic shoulder pain of unspecific origin.

Chronic shoulder pain is a complex painful condition, with no clear clinical definition [3], whose high recurrence and persistence of symptoms [1, 2], make it difficult to choose the most appropriate treatment. Also a relationship has been shown between high prevalence of myofascial trigger points in the shoulder muscles and the presence of pain [1, 3, 13, 14], so these patients could benefit from an approach focused on muscle treatment.

Moreover, previous studies have shown the efficacy of the combination of manual therapy techniques and therapeutic exercise for shoulder pain, although the best frequency and dosing are not clear [5, 17]. The use of dry needling is recommended in patients with Myofascial Pain Syndrome of the Upper Quadrant [4], while the benefits of a single session of dry needling in a multimodal programme has been observed in cases of post-surgical shoulder pain [19].

Moreover, the benefits of therapeutic exercises in cases of shoulder pain [26, 36], show the importance of including them in the implementation of this protocol, not only during treatment sessions but also at home. Among the exercises included in this protocol, scapular training is a fundamental aspect, as a reduction in electromyographic activation in the serratus anterior and lower trapezius has been observed in patients with shoulder pain, as well as greater activation of the upper trapezius, reflected in a scapulohumeral muscle imbalance [36].

Thus, and based on the literature, we selected different techniques of manual physiotherapy and therapeutic exercise for the creation of this intervention protocol, which also includes a single session of dry needling or sham dry needling to study its potential benefits. Being techniques with few adverse effects and being cheaper in terms of cost-effectiveness, it may be an alternative to more aggressive interventions such as surgery and infiltrations for which similar short-term results have been obtained [51].

Therefore, due to the lack of similar studies for patients with chronic shoulder pain of unspecified origin, the implementation of this study and publication of the results will make a new contribution in the field of chronic shoulder pain treatment, and may establish new research lines in which the effects of dry needling in chronic shoulder pain can be studied and the best frequency and dosage can be established.

## Abbreviations

DASHe: Disabilities of Arm, Shoulder and Hand Spanish version
ICC: Intraclass correlation coefficient
MTrPs: Myofascial trigger points
NSAIDs: Anti-inflammatory drugs
PPT: Pressure pain threshold
SD: Standard deviation
VAS: Visual analogue scale

## References

[1] Bron C, de Gast A, Dommerholt J, Stegenga B, Wensing M, Oostendorp RA (2011). Treatment of myofascial trigger points in patients with chronic shoulder pain: a randomized, controlled trial. BMC Med.

[2] Worsley P, Warner M, Mottram S (2013). Motor control retraining exercises for shoulder impingement: effects on function, muscle activation, and biomechanics in young adults. J Shoulder Elb Surg.

[3] Sergienko S, Kalichman L (2015). Myofascial origin of shoulder pain: a literature review. J Bodyw Mov Ther.

[4] Peek AL, Miller C, Heneghan NR (2015). Thoracic manual therapy in the management of non-specific shoulder pain: a systematic review. J Man Manip Ther.

[5] Kietrys DM, Palombaro KM, Azzaretto E (2013). Effectiveness of dry needling for upper-quarter myofascial pain: a systematic review and meta-analysis. J Orthop Sport Phys.

[6] Pope DP, Croft PR, Pritchard CM, Silman AJ (1997). Prevalence of shoulder pain in the community: the influence of case definition. Ann Rheum Dis.

[7] Farrar JT, Young JP, LaMoreaux L, Werth JL, Poole RM (2001). Clinical importance of changes in chronic pain intensity measured on an 11-point numerical pain rating scale. Pain.

[8] Kalichman L, Vulfsons S (2010). Dry needling in the management of musculoskeletal pain. J Am Board Fam Med.

[9] Skootsky SA, Jaeger B, Oye RK (1989). Prevalence of myofascial pain in general internal medicine practice. West J Med.

[10] Schibany N, Zehetgruber H, Kainberger F (2004). Rotator cuff tears in asymptomatic individuals: A clinical and ultrasonographic screening study. Eur J Radiol.

[11] Needell SD, Zlatkin MB, Sher JS, Murphy BJ, Uribe JW (1996). MR imaging of the rotator cuff: Peritendinous and bone abnormalities in an asymptomatic population. AJR Am J Roentgenol.

[12] Naranjo A, Marrero-Pulido T, Ojeda S (2002). Abnormal sonographic findings in the asymptomatic arthritic shoulder. Scand J Rheumatol.

[13] Simons DG, Travell JG, Simons LS. Travell & simons’ myofascial pain and dysfunction: upper half of body. Vol 1. USA: Lippincott Williams & Wilkins; 1999.

[14] Bron C, Dommerholt J, Stegenga B, Wensing M, Oostendorp RA (2011). High prevalence of shoulder girdle muscles with myofascial trigger points in patients with shoulder pain. BMC Musculoskel Dis.

[15] Morrison DS, Frogameni AD, Woodworth P (1997). Non-operative treatment of subacromial impingement syndrome. J Bone Joint Surg Am.

[16] Wang JC, Horner G, Brown ED, Shapiro MS (2000). The relationship between acromial morphology and conservative treatment of patients with impingement syndrome. Orthopedics.

[17] Camarinos J, Marinko L (2009). Effectiveness of manual physical therapy for painful shoulder conditions: a systematic review. J Man Manip Ther.

[18] Ge H, Fernández-de-las-Peñas C, Madeleine P, Arendt-Nielsen L (2008). Topographical mapping and mechanical pain sensitivity of myofascial trigger points in the infraspinatus muscle. Eur J Pain.

[19] Arias-Buría JL, Valero-Alcaide R, Cleland JA (2015). Inclusion of trigger point dry needling in a multimodal physical therapy program for postoperative shoulder pain: a randomized clinical trial. J Manip Physiol Ther.

[20] Gerwin RD, Shannon S, Hong C, Hubbard D, Gevirtz R (1997). Interrater reliability in myofascial trigger point examination. Pain.

[21] Cohen J (1988). Statistical power analysis for the behavioral sciences.

[22] Faul F, Erdfelder E, Lang A, Buchner A (2007). G* power 3: a flexible statistical power analysis program for the social, behavioral, and biomedical sciences. Behav Res Methods.

[23] Hong C (2006). Treatment of myofascial pain syndrome. Curr Pain Headache Rep.

[24] Hong C (1994). Lidocaine injection versus dry needling to myofascial trigger point: the importance of the local twitch response. Am J Phys Med Rehabil.

[25] Sterling M, Vicenzino B, Souvlis T, Connelly LB (2015). Dry-needling and exercise for chronic whiplash-associated disorders: A randomized single-blind placebo-controlled trial. Pain.

[26] Kuhn JE (2009). Exercise in the treatment of rotator cuff impingement: a systematic review and a synthesized evidence-based rehabilitation protocol. J Shoulder Elb Surg.

[27] Tautenhahn UG, de Bie RA, Staal JB, Bastiaenen CH, Kromer TO (2009). Effects of physiotherapy in patients with shoulder impingement syndrome: a systematic review of the literature. J Rehabil Med.

[28] Hidalgo-Lozano A, Fernández-de-las-Peñas C, Díaz-Rodríguez L, González-Iglesias J, Palacios-Ceña D, Arroyo-Morales M (2011). Changes in pain and pressure pain sensitivity after manual treatment of active trigger points in patients with unilateral shoulder impingement: a case series. J Bodywork Movement Ther.

[29] Bennell K, Wee E, Coburn S (2010). Efficacy of standardised manual therapy and home exercise programme for chronic rotator cuff disease: randomised placebo controlled trial. BMJ.

[30] Kachingwe AF, Phillips B, Sletten E, Plunkett SW (2008). Comparison of manual therapy techniques with therapeutic exercise in the treatment of shoulder impingement: a randomized controlled pilot clinical trial. J Man Manip Ther.

[31] Johnson AJ, Godges JJ, Zimmerman GJ, Ounanian LL (2007). The effect of anterior versus posterior glide joint mobilization on external rotation range of motion in patients with shoulder adhesive capsulitis. J Orthop Sports Phys Ther.

[32] Yang JL, Chang CW, Chen SY, Wang SF, Lin JJ (2007). Mobilization techniques in subjects with frozen shoulder syndrome: randomized multiple-treatment trial. Phys Ther.

[33] Vermeulen HM, Rozing PM, Obermann WR, le Cessie S, Vliet Vlieland TP (2006). Comparison of high-grade and low-grade mobilization techniques in the management of adhesive capsulitis of the shoulder: Randomized controlled trial. Phys Ther.

[34] Delgado-Gil JA, Prado-Robles E, Rodrigues-de-Souza DP, Cleland JA, Fernández-de-las-Peñas C, Alburquerque-Sendín F. Effects of mobilization with movement on pain and range of motion in patients with unilateral shoulder impingement syndrome: a randomized controlled trial. J Manip Physiol Ther. 2015;38(4):245-252. 10.1016/j.jmpt.2014.12.008 25936465

[35] Teys P, Bisset L, Vicenzino B (2008). The initial effects of a mulligan’s mobilization with movement technique on range of movement and pressure pain threshold in pain-limited shoulders. Man Ther.

[36] Hanratty CE, McVeigh JG, Kerr DP (2012). The effectiveness of physiotherapy exercises in subacromial impingement syndrome: a systematic review and meta-analysis. Sem Arthritis Rheu.

[37] Ellenbecker TS, Cools A (2010). Rehabilitation of shoulder impingement syndrome and rotator cuff injuries: an evidence-based review. Br J Sports Med.

[38] Dun S, Barrentine SW, Ellerbusch MT, Andrews JR (2007). Electromyographic analysis of the supraspinatus and deltoid muscles during 3 common rehabilitation exercises. J Athl Train.

[39] Struyf F, Nijs J, Mollekens S (2013). Scapular-focused treatment in patients with shoulder impingement syndrome: A randomized clinical trial. Clin Rheumatol.

[40] Ortega-Castillo M, Medina-Porqueres I (2016). Effectiveness of the eccentric exercise therapy in physically active adults with symptomatic shoulder impingement or lateral epicondylar tendinopathy: a systematic review. J Sci Med Sport.

[41] Gallagher EJ, Liebman M, Bijur PE (2001). Prospective validation of clinically important changes in pain severity measured on a visual analog scale. Ann Emerg Med.

[42] Bird SB, Dickson EW (2001). Clinically significant changes in pain along the visual analog scale. Ann Emerg Med.

[43] Tashjian RZ, Deloach J, Porucznik CA, Powell AP (2009). Minimal clinically important differences (MCID) and patient acceptable symptomatic state (PASS) for visual analog scales (VAS) measuring pain in patients treated for rotator cuff disease. J Shoulder Elb Surg.

[44] Hervás MT, Collado MJN, Peiró S, Pérez JLR, Matéu PL, Tello IM (2006). Versión española del cuestionario DASH. adaptación transcultural, fiabilidad, validez y sensibilidad a los cambios. Med Clin.

[45] Schmitt JS, Di Fabio RP (2004). Reliable change and minimum important difference (MID) proportions facilitated group responsiveness comparisons using individual threshold criteria. J Clin Epidemiol.

[46] Vanderweeen L, Oostendorp R, Vaes P, Duquet W (1996). Pressure algometry in manual therapy. Man Ther.

[47] Chesterton LS, Sim J, Wright CC, Foster NE (2007). Interrater reliability of algometry in measuring pressure pain thresholds in healthy humans, using multiple raters. Clin J Pain.

[48] Cools AM, De Wilde L, Van Tongel A, Ceyssens C, Ryckewaert R, Cambier DC (2014). Measuring shoulder external and internal rotation strength and range of motion: Comprehensive intra-rater and inter-rater reliability study of several testing protocols. J Shoulder Elb Surg.

[49] Andersen KS, Christensen BH, Samani A, Madeleine P (2014). Between-day reliability of a hand-held dynamometer and surface electromyography recordings during isometric submaximal contractions in different shoulder positions. J Electromyogr Kinesiol.

[50] Douma RK, Soer R, Krijnen WP, Reneman M, van der Schans, Cees P (2014). Reference values for isometric muscle force among workers for the netherlands: a comparison of reference values. BMC Sports Sci Med Rehabil.

[51] Liu L, Huang Q, Liu Q, et al. Effectiveness of dry needling for myofascial trigger points associated with neck and shoulder pain: a systematic review and meta-analysis. Arch Phys Med Rehabil. 2015;96(5):944-55. 10.1016/j.apmr.2014.12.015 25576642

